# Brain Networks Responsible for Sense of Agency: An EEG Study

**DOI:** 10.1371/journal.pone.0135261

**Published:** 2015-08-13

**Authors:** Suk Yun Kang, Chang-Hwan Im, Miseon Shim, Fatta B. Nahab, Jihye Park, Do-Won Kim, John Kakareka, Nathanial Miletta, Mark Hallett

**Affiliations:** 1 Department of Neurology, Dongtan Sacred Heart Hospital, Hallym University College of Medicine, Hwaseong Si, Gyeonggi-Do, Republic of Korea; 2 Human Motor Control Section, National Institute of Neurological Disorders and Stroke, National Institutes of Health, Bethesda, Maryland, United States of America; 3 Department of Biomedical Engineering, Hanyang University, Seoul, Republic of Korea; 4 Department of Neurology, University of California San Diego, San Diego, California, United States of America; 5 Department of Biomedical Engineering, Yonsei University, Wonju-si, Kangwon-do, Republic of Korea; 6 Signal Processing and Instrumentation Section, Division of Computational Bioscience, Center for Information Technology, National Institutes of Health, Bethesda, Maryland, United States of America; Interdisciplinary Center (IDC) Herzliya, ISRAEL

## Abstract

**Background:**

Self-agency (SA) is a person’s feeling that his action was generated by himself. The neural substrates of SA have been investigated in many neuroimaging studies, but the functional connectivity of identified regions has rarely been investigated. The goal of this study is to investigate the neural network related to SA.

**Methods:**

SA of hand movements was modulated with virtual reality. We examined the cortical network relating to SA modulation with electroencephalography (EEG) power spectrum and phase coherence of alpha, beta, and gamma frequency bands in 16 right-handed, healthy volunteers.

**Results:**

In the alpha band, significant relative power changes and phase coherence of alpha band were associated with SA modulation. The relative power decrease over the central, bilateral parietal, and right temporal regions (C4, Pz, P3, P4, T6) became larger as participants more effectively controlled the virtual hand movements. The phase coherence of the alpha band within frontal areas (F7-FP2, F7-Fz) was directly related to changes in SA. The functional connectivity was lower as the participants felt that they could control their virtual hand. In the other frequency bands, significant phase coherences were observed in the frontal (or central) to parietal, temporal, and occipital regions during SA modulation (Fz-O1, F3-O1, Cz-O1, C3-T4L in beta band; FP1-T6, FP1-O2, F7-T4L, F8-Cz in gamma band).

**Conclusions:**

Our study suggests that alpha band activity may be the main neural oscillation of SA, which suggests that the neural network within the anterior frontal area may be important in the generation of SA.

## Introduction

Sense of agency, specifically self-agency (SA), is the sense that “I am the one who is causing or generating an action.”[1]. It includes a spontaneous feeling that “self” is distinguished from “others” in the outside world during action generation [2]. SA is associated with the effect-related aspect of free will [3,4]. It is an essential component of voluntary movement allowing us to feel that we own our actions and that our actions are voluntary. The sense of agency derives from the proper match of volition and movement feedback [4]. The sense of ownership is a different concept, the sense that I am the one who is undergoing an experience [1]. For example, patients with involuntary movement disorders do not feel SA, but acknowledge their body is moving (intact sense of ownership).

Disturbances of SA arise in many neurological and mental disorders such as alien limb syndrome, psychogenic movement disorders, and schizophrenia [3,4]. Patients with these disorders deny their actions are voluntary. Researchers have tried to measure SA in human voluntary behavior, including various neurological and psychiatric diseases.

Recent neuroimaging studies identified several areas (ventral premotor cortex, supplementary motor area, cerebellum, dorsolateral prefrontal cortex, posterior parietal cortex, posterior segment of the superior temporal sulcus, insula, and extrastriate body area, particularly in the right hemisphere) relating to SA [2,5–10]. Only a small number of studies investigated the correlation between the amount of brain activity and the amount of SA. Generally, the frontal areas such as the insula showed higher activation when participants felt that they could control their movements more, whereas the inferior parietal cortex showed increased activation when they felt they were controlling less [5,11]. Most previous studies assessed the mismatch between intended action and real outcomes. Ecologically valid virtual reality applications may generate SA properly and are therefore useful to study [10,12].

SA is associated with a number of brain areas because of many linked factors and it may be difficult to extract what each exactly does. Understanding the interrelationship of these responsible areas may shed light on the mechanism of SA. Functional connectivity is believed to afford the physiological basis for information processing and mental representation [13]. Since fMRI shows localized changes in detail, it is a major method in cognitive science research [14]. Our lab previously reported two discrete networks (leading and lagging in a 30-second scanning block) using fMRI, inferring a spatial and temporal flow of information in SA. The “leading regions” (activated early in the scanning block) consisting of the right supramarginal gyrus, left anterior inferior parietal lobule, anterior insula, and right temporoparietal junction might be involved in mismatch identification. The “lagging regions” (activated later in the block) of the bilateral prefrontal, cingulate, and bilateral posterior inferior parietal lobule might receive this mismatched information and generate SA [10].

Neural oscillations are considered to mediate neural activity in functional networks [15]. Although real-time fMRI analysis now allows investigating functional connectivity networks between different brain areas [16], it may have technical limitations. fMRI, using blood-oxygen-level dependent (BOLD), is an indirect measurement of neuronal activity. Another concern comes from whether the slow activity (<0.1 Hz) in fMRI is actually correlated with fast neuronal activity (1–80 Hz) in EEG [17]. EEG can provide high temporal resolution information about brain dynamics and direct functional connectivity between SA-associated areas [16].

To find out the cortical network relating to SA, we conducted EEG research with an ecologically valid virtual reality application used in a previous fMRI study [10]. To estimate neuronal activity and functional connectivity, we used EEG power spectrum measures and phase coherence. Because no prior studies investigated the functional network properties of SA using EEG, we selected candidate frequencies of EEG based on previous studies of execution and motion observation [18–27]. We tested the hypothesis that alpha, beta, and gamma band activities over frontal, parietal and temporal regions would be directly correlated with SA. More specifically, we hypothesized that lower relative power decreases relating to SA would be associated with better self-control of the motor task. We thought that the functional connectivity between frontal and parietal areas would be correlated with SA because sensorimotor information (parietal) should be judged by supervisory areas (frontal) [2]. We hypothesized that phase coherence would be more increased with less self-control, because more of the neuronal population would be synchronized to react to this altered SA (i.e., unusual situation).

## Materials and Methods

### Participants

Nineteen right-handed, healthy volunteers (13 men, 6 women, mean age 28±4.2 years, range 22–35 years) participated in the EEG experiment. We recruited them from the National Institutes of Health (NIH) database population and local community. General medical screening, neurological examination, and a routine MRI were done. Participants were excluded if they had any abnormal findings on neurological examination, any history of brain tumor, stroke, head trauma or a vascular malformation, any structural lesions from the routine MRI studies. They were also excluded if their medical condition was not proper for EEG recordings. Participants were also instructed to abstain from caffeine and alcohol for 48 h prior to recording. All were naïve to this experiment. We did not enroll anyone who had participated in the previous fMRI study in our lab [10]. The data of 16 participants (10 men, mean age 27.0± 4.0 years) were analyzed. We excluded 3 participants (3 men, mean age 31.3± 4.7 years) from the analysis because of technical problems during data acquisition. All participants had normal or corrected-to-normal vision. Before the start of the EEG experiment, all participants gave written informed consent for this research protocol, which was approved by the National Institutes of Health (NIH) Institutional Review Board.

### Overall procedures

Overall study design was nearly identical to the previous SA experiment using fMRI in our lab (for more detailed description on the experimental condition, see [10]). Before EEG recordings, all participants were fitted with a CyberGlove (CyberGlove Systems LLC, San Jose, CA) on their right hands. Data from finger movements were recorded and could be used to control the image of a hand on a computer monitor. The glove was calibrated so that real hand movements could be correctly represented on a computer monitor. After calibrating to ensure comfort, participants were asked to flex the individual fingers of their right hand one-by-one while watching their virtual hand on the monitor. They were asked to perform two different tasks: (1) On the emergence of the virtual hand on the monitor, participants were asked to move their fingers with their own free will and with visual feedback. The virtual hand variably imitated the movement of the fingers. A computerized program combined different amounts of their movements with random movements introduced by the computer. There were five levels of modulation: 0%, 25%, 50%, 75%, and 100%. 0% control meant that participants were not controlling the virtual hand at all. 100% control indicated that participants were in total control of the virtual hand (that is, the virtual hand completely mimicked the participant’s hand movements). 25%, 50%, and 75% signified intermediate levels of control. (2) When a “+” appeared on the monitor, participants were asked to watch the “+” without hand movements (called “+” condition). The sequence of the two tasks (“+” condition, five levels of % control condition) was pseudo-randomly arranged in block design. Each % control and “+” condition was displayed five times. Each EEG block was 5 min. The duration of each % control and “+” condition was 20 sec. The participants practiced the tasks twice prior to the EEG recording.

During EEG recording, participants sat on a comfortable chair in a quiet, dimly-lit room. Dual monitors were set up and duplicated their virtual hands. A 21-inch monitor in front of the participants was located at a distance of 2 m from their nasion. Participants were instructed to keep their eyes open and to fixate on the images on the monitor during the entire recording to reduce irrelevant ocular movement and blinking. The center of the monitor was adjusted to the participant’s eye level. Through another monitor, experimenters could operate the virtual hand task program and see the virtual hand movement of the participants. Participants could see their virtual hands, but not their real hands. The background of the virtual hands was black. They performed the two different tasks according to the monitor displaying the images. The hand movements were individual finger flexions and extensions beginning from the fifth finger to the second finger and reversing. Participants were asked to decide the speed of their finger movements at their own choice before EEG recording and to keep the same speed during EEG recording. This was done because we assumed that their own decisions might enhance the cerebral activity of SA. However, to maintain their speed consistency across the study, we also instructed them not to move either too fast or too slow, because the difference of the speed within each individual and among participants might make cerebral activity different, which could bias our results [28]. With the instructions of “not too fast or not too slow” led to finger velocities being in a similar range. We also monitored the finger movements in real time. In the case of real hand movements with visual feedback, we gave information that sometimes the virtual hand on the monitor would not follow correctly and asked participants to keep pace with their real hand movements. This instruction was for participants to feel themselves generating virtual hand movements and not to follow the virtual hand movements on the monitor.

After finishing the EEG recording, we removed the EEG electrodes and asked participants to sit in the comfortable chair again and to perform the finger movements with visual feedback again. There were five levels of % control. Each level of control of the visual hand, pseudo-randomly arranged, was seen twice (total 10 blocks). They were asked to rate their feeling of controlling their virtual hand right after they performed each level of task. They could report any number from 0% to 100%. We correlated the actual level of control with the level of control that participants subjectively perceived.

### Data acquisition and preprocessing

EEG data were recorded from 58 tin EEG electrodes (FPZ, FP1, F3A, Fz, F1, F3, F5, F7, CzA, C1A, C3A, C5A, Cz, C1, C3, C5, T3, FP2, F4A, F2, F4, F6, F8, C2A, C4A, C6A, C2, C4, C6, T4, C1P, C3P, TCP1, T3L, Pz, P1, P3, P5, PzA, T5, PzP, P1P, P3P, Oz, O1, CB1, C2P, C4P, TCP2, T4L, P2, P4, P6, T6, P2P, P4P, O2, CB2) mounted on a cap (Electro-Cap International, Inc., Eaton, OH) according to the international 10–20 system with a right earlobe reference. EEG data were later converted to digitally-linked earlobe reference. EEG data were acquired using SynAmps amplifiers (Compumedics, El Paso, TX) at a sampling rate of 1 kHz and bandpass filtered from DC to 100 Hz. An electro-oculogram and surface electromyogram (EMG) from the extensor carpi radialis (ECR) and flexor carpi ulnaris (FCU) muscles of both arms with a bandpass range of 5–200 Hz were recorded. Bipolar recordings of the vertical and horizontal electro-oculogram were done. Surface EMG on the right arm was used to detect the time of onset and termination of movement; surface EMG on the left side was to monitor any movement during EEG recording.

The raw EEG data were initially processed using Scan 4.3 (Compumedics, El Paso, TX). Gross movement artifacts which were considered unrelated to hand movements were removed by visual inspection. After artifact rejection, the continuous EEG data were epoched into 1-sec segments from the beginning of each trial (a 20-sec task period). Any epoch containing significant physiological artifacts, amplitude exceeding ±75 μV, were also excluded from the analysis. Among the 50 trials of each condition, the numbers of remaining epochs were 36.81 ± 7.84 for 0% condition, 34.62 ± 9.00 for 25% condition, 38.38 ± 6.97 for 50% condition, 37.75 ± 6.19 for 75% condition and 29.81 ± 6.16 for 100% conditions.

### Data analysis

We first evaluated the spectral power at each electrode. The spectral power of each 1-sec epoch was computed by applying short-time Fourier transform (STFT) with a 512 point sliding Hanning window and 50% overlap. The result was then averaged across trials for each condition and each electrode. The averaged spectral power of each condition and electrode was then accumulated over three frequency bands; alpha (8–12 Hz), beta (13–30 Hz), and gamma (31–50 Hz). For further statistical analysis, the relative power between each condition and resting condition (“+” condition) was calculated in decibels, which was plotted in a topographical map.

To compare the synchronization between the electrodes with respect to different conditions, we computed phase coherence for each electrode pairs, which can determine the degree of synchronization between two time series and is calculated from:

$$ERPCOH^{a,b}(f,t)=\frac{1}{n}\overset{n}{\underset{k=1}{\sum }}\frac{F_{k}^{a}(f,t)F_{k}^{b}{\left(f,t\right)}^{*}}{\left|F_{k}^{a}(f,t)F_{k}^{b}(f,t)\right|}$$

where *f* and *t* represent frequency and time, respectively; superscripts *a* and *b* indicate two selected channels; and *n* is the number of epochs. $F_{k}^{a}\left(f,t\right)$ was calculated by short-time Fourier transform [29]. $F_{k}^{b}{\left(f,t\right)}^{*}$ represents the complex conjugate of $F_{k}^{b}\left(f,t\right)$ and | | operator represents the complex norm. The result has a minimum value of 0 to a maximum value of 1, where 0 indicates complete absence of synchronization between two signals, *a* and, *b* at a given frequency *f* in the time window centered at *t*, and 1 indicates perfect synchronization. The phase coherence values were then averaged across trials for each condition and each electrode pairs.

**Fig 1:**
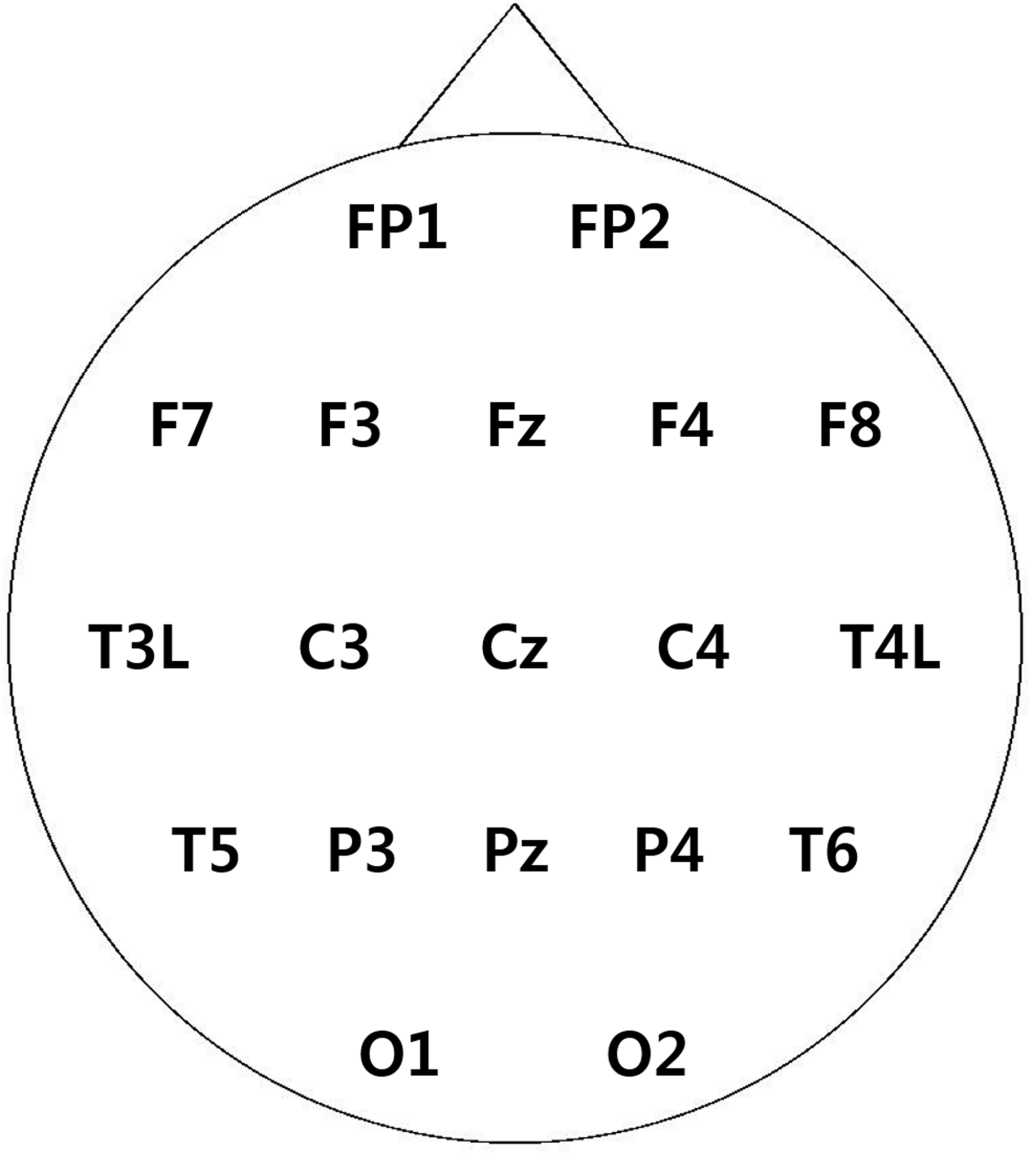
The location of all recording electrodes in the international 10–20 EEG system.

Considering the computational cost and for the sake of simplicity, we chose 19 electrodes (FP1, FP2, Fz, F3, F4, F7, F8, Cz, C3, C4, T3L, T4L, Pz, P3, P4, T5, T6, O1, O2) according to the traditional 10–20 system from 58 channels (Fig 1). Since contamination of EEG signals was observed at the T3 and T4 electrodes in a few participants, those channels were replaced with adjacent channels (T3L and T4L). In this study, the power spectrum and phase coherence were calculated using functions implemented in a MATLAB toolbox EEGLAB (http://sccn.ucsd.edu/eeglab/).

### Statistical analysis

Because we thought that the cerebral activity relating to SA should differ from the resting cerebral activity, and if not, it might be irrelevant in spite of any significant differences in % control conditions, we first compared the each % condition with the resting condition (“+” condition). The frequency-specific relative power changes over SA modulation were analyzed. We first selected channels in each frequency band showing significant spectral power increments or decrements in at least one of the five % control conditions, compared to the resting condition, “+” condition (Wilcoxon signed-rank test, p < 0.0003, Bonferroni corrected). Any electrode with at least one significant frequency band was selected for the further analyses (S1 Table). After we selected the electrodes and frequency bands with statistically significant power changes, a follow-up statistical test was performed to compare the relative power among the five % control conditions using Friedman test (Bonferroni corrected p-values were used for pair-wise post-hoc analysis). Relative power means the average baseline (i.e., resting condition) subtracted values from the average spectral power for each participant.

Similarly, we first selected electrode pairs with significant differences in connectivity between each band and each condition compared to resting condition. Comparisons were done for the all possible pairs of 19 individual electrodes using Wilcoxon signed-rank test (p<0.0003, Bonferroni corrected). Any electrode pair with significant difference in at least one of the five % control conditions for each band was selected (S2 Table). After the statistical analysis, the lines significantly different from fixation condition were represented by line color (red indicate the phase coherence increases and blue indicate the phase coherence decreases). To compare the differences in connectivity strength among five % control conditions, the phase coherence values were tested with Friedman test (Bonferroni corrected p-values were used for pair-wise post-hoc analysis) for each selected pair of electrodes. The statistical analyses were calculated using MATLAB 2009a (Mathworks, Inc., USA) and SPSS 17.

## Results

### Behavioral Measurements

**Fig 2:**
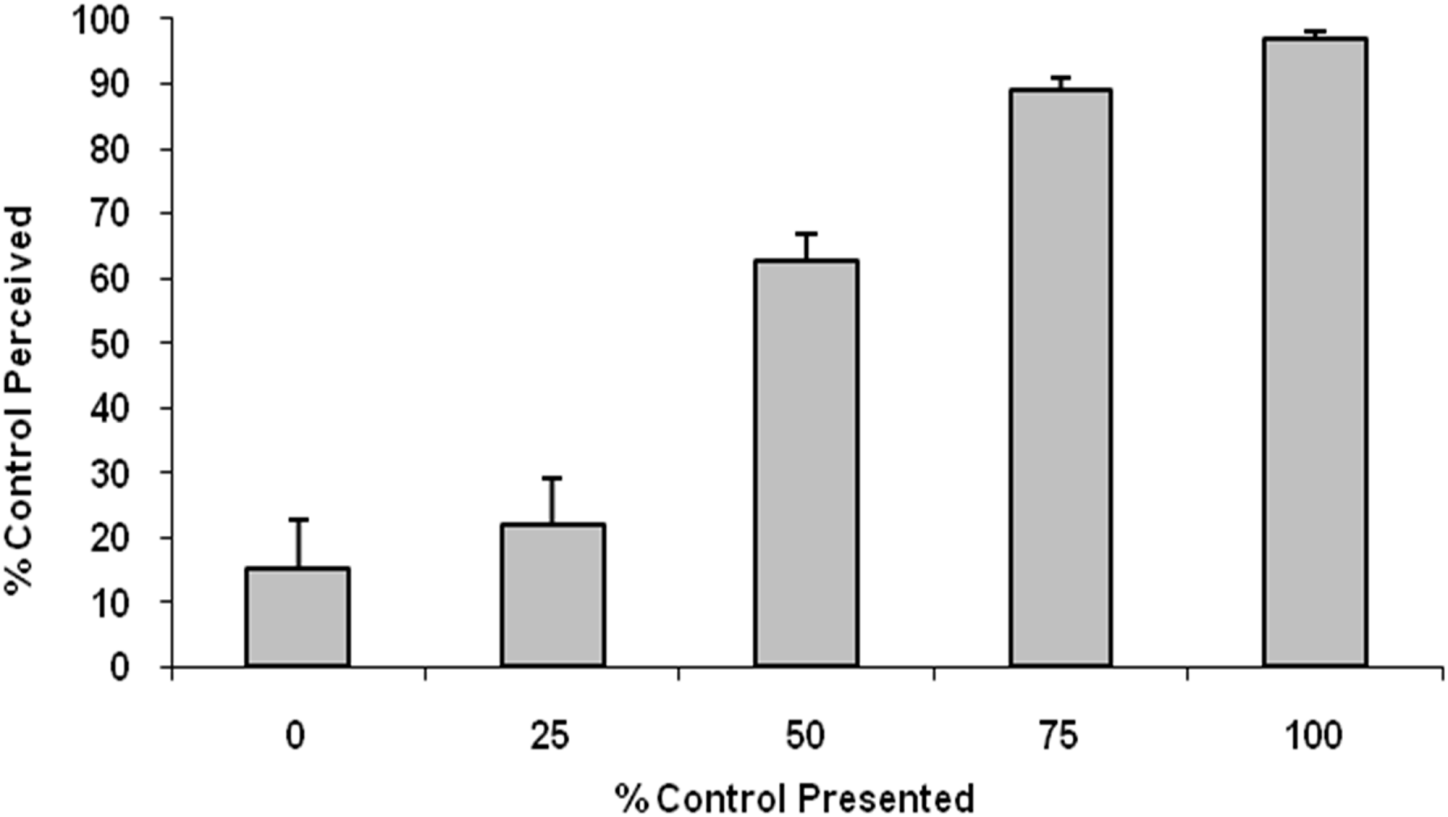
Behavioral data of visual feedback of virtual hand movements in 16 participants. The x-axis indicates the degree of % control of virtual hand movements in the monitor. The y-axis indicates the value (mean ± SEM) of % control that participants report (i.e., subjective feelings). Perception of control is well correlated with the presentation level of the virtual hands in the monitor. These results are consistent with a previous study in our lab (Nahab et al., 2011).

The perceived % controls of 16 participants correlated well with the % controls that were presented on the monitor (Fig 2). These results were consistent with the previous study in our lab [10]. The level of % control was overrated at 50% and 75% control, 62.5±17.6% (mean ±SD) and 89.2±6.9%, respectively (*p* = 0.013, *p*<0.001, respectively; one-sample t-test). The level of % control was slightly underrated at the 100% level (96.9±4.5%, *p* = 0.015; one-sample t-test). Participants overestimated 0% control (15.3±29.6%) and underestimated 25% control (21.8±29.1%), but there was no significant difference. Standard deviations of the mean value of the reported SA became smaller as the level of actual % control increased. This means there were smaller inter-individual variations at the higher levels of the presented % control.

### Frequency-specific power changes with SA modulation

**Fig 3:**
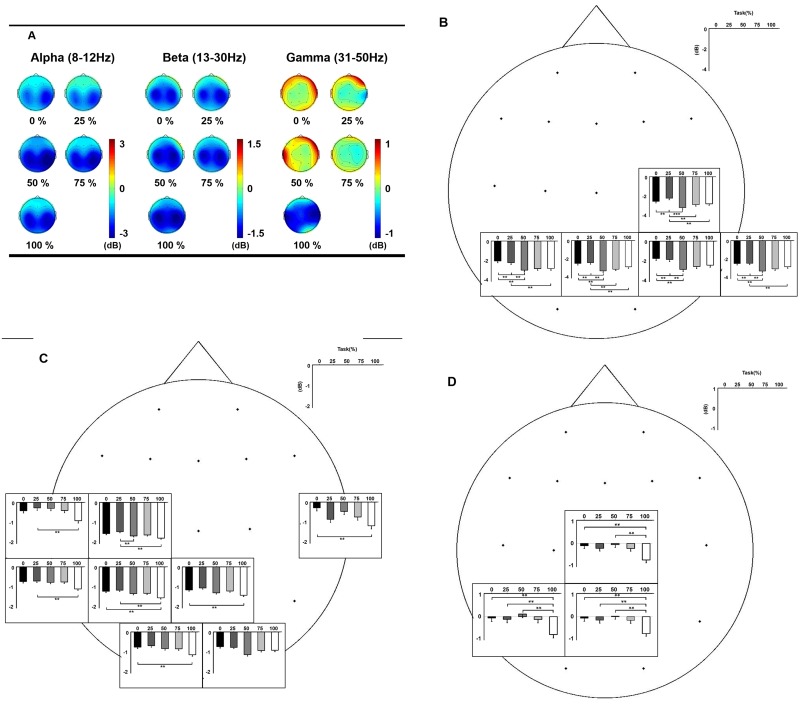
Frequency-specific relative power changes over SA modulation. Each frequency band represents the group-averaged, relative power spectrum. (A) Topographic maps according to % control conditions. Higher number of the % control indicates better control of the subjects’ virtual hands (0% control, no control; 100% control, complete control). Blue colors indicate relative power decreases and red colors indicate power increases. (B) alpha (8–12 Hz) band. (C) beta (13–30 Hz) band. (D) gamma (31–50 Hz) band. In each frequency band, the bar charts in the rectangles show the changes of relative powers over sense of agency (SA) modulation in the corresponding electrodes of the individual rectangles. The insets at each electrode location are the ones with significant power changes using Friedman test. The dots indicate electrode positions. Overall, alpha bands are closely related to SA changes, compared with other frequency bands. The relative power changes over SA changes are significant in the central, bilateral parietal, and right temporal regions showing the relative power decreases are bigger at the higher level of % control. The asterisks indicate significant differences between the pairs of % control conditions (Bonferroni corrected p-values for pair-wise post-hoc analysis). *p<0.05, **p<0.01, ***p<0.001.

In general, of the three frequency bands, the alpha and beta bands were most closely associated with SA modulation. The most prominent relative power decreases of EEG were observed in the alpha band (Fig 3A). Significant relative power changes with SA modulation were in the central, parietal and temporal areas; relative power decreases were more prominent with a higher level of % control (Fig 3B–3D).

In the alpha band, significant relative power changes were observed as the level of SA was modulated in the central, bilateral parietal, and right temporal areas (C4: *χ* ^2^ = 21.85, *p* = 0.0002; Pz: *χ* ^2^ = 21.2, *p* = 0.0003; P3: *χ* ^2^ = 14.25, *p* = 0.007; P4: *χ* ^2^ = 18.55, *p* = 0.0009; T6: *χ* ^2^ = 17.45, *p* = 0.002; Friedman test). The magnitude of the relative power decrease was larger at the higher level of control (50%, 75%, 100% control condition), but it was not linear. The largest decrease was at the 50% control condition, but there was no significant difference among 50%, 75%, and 100% control condition.

Relative power decrease of beta band activity was in the central, left sensorimotor, bilateral temporal, and bilateral occipital areas (C3: *χ* ^2^ = 10.40, *p* = 0.021; T3L: *χ* ^2^ = 11.60, *p* = 0.020; Pz: *χ* ^2^ = 11.30, *p* = 0.023; P3: *χ* ^2^ = 13.35, *p* = 0.009; T5: *χ* ^2^ = 11.65, *p* = 0.020; O1: *χ* ^2^ = 15.65, *p* = 0.003; T4L: *χ* ^2^ = 10.05, *p* = 0.039; O2: *χ* ^2^ = 10.65, *p* = 0.031; Friedman test). It was largest at 100% control condition and similar among the other control conditions (0%, 25%, 50%, 75% control condition).

In the gamma band, significant relative power changes were found in the central (Cz: *χ* ^2^ = 15.10, *p* = 0.004; Pz: *χ* ^2^ = 17.75, *p* = 0.001; Friedman test) and the left parietal areas (P3: *χ* ^2^ = 20.65, *p* = 0.0003; Friedman test) according to the level of SA. The relative power decrease was greatest at 100% control condition, which was statistically significant in the other % control (0%, 25%, 50%, 75%) conditions (Fig 3D). There was no significant change in the other control conditions.

### Frequency-specific phase coherence changes over SA modulation

**Fig 4:**
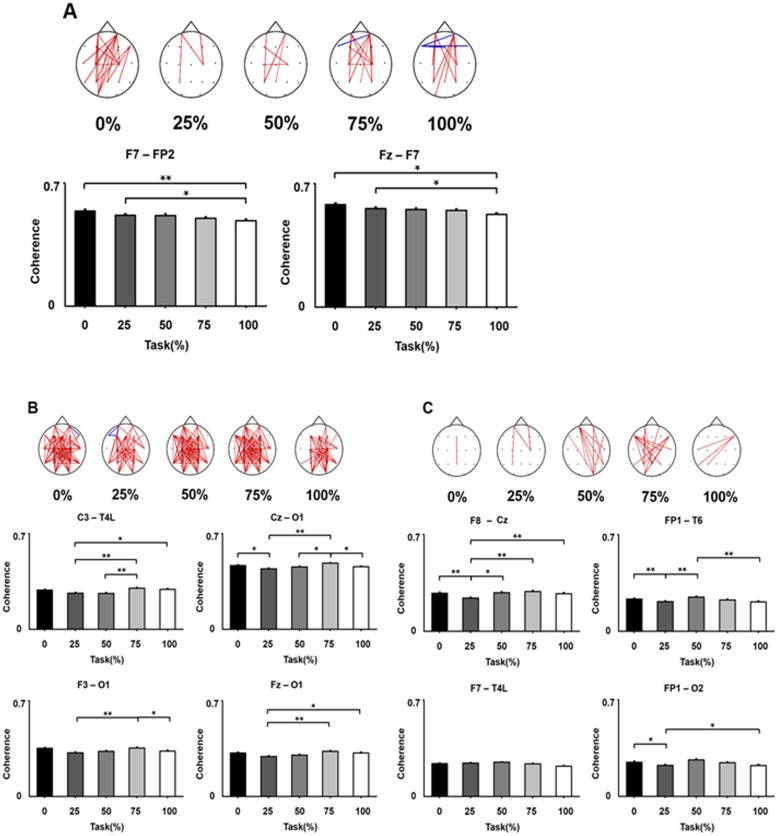
Frequency-specific phase coherence changes over SA modulation. (A) alpha (8–12 Hz) band. (B) beta (13–30 Hz) band. (C) gamma (31–50 Hz) band. In each frequency band, topographic maps show group-averaged, phase coherence. The dots indicate electrode positions. Red lines indicate significant phase coherence increases and blue lines indicate significant phase coherence decreases, compared to the value of phase coherence in the resting condition. Graphs represent the pairs of electrodes showing significant phase coherence changes according to SA modulation. The bar charts show the electrode pairs with significant variations of phase coherence according to SA changes in three frequency bands (alpha, beta, gamma). The asterisks indicate significant differences between the pairs of % control condition (Bonferroni corrected p-values for pair-wise post-hoc analysis). *p<0.05, **p<0.01.

Overall, the phase coherence changes in the three frequency bands (alpha, beta, and gamma) seemed to be associated with SA modulation. Among them, the alpha band was most closely correlated with SA modulation (Fig 4A). The significant changes of the alpha band phase coherence with SA modulation were within the anterior frontal regions (Fig 4).

In the alpha band, two pairs of electrodes showed significant changes in phase coherence according to SA modulation: left and right frontal (F7-FP2: *χ* ^2^ = 18.80, *p* = 0.0008; Friedman test) and left frontal and mid-central (F7-Fz: *χ* ^2^ = 9.95, *p* = 0.041; Friedman test) areas. Synchronization of phase coherence became significantly smaller at higher levels of % control condition. (Fig 4A).

In the beta band, phase coherence was significantly changed on the frontal and right occipital (Fz-O1: *χ* ^2^ = 9.65, *p* = 0.046; F3-O1: *χ* ^2^ = 9.90, *p* = 0.042; Friedman test), the mid-central and right occipital (Cz-O1: *χ* ^2^ = 12.05, p = 0.016; Friedman test), and the left central and the right temporal area (C3-T4L: *χ* ^2^ = 11.4, p = 0.022; Friedman test) over SA modulation. Unlike the alpha band, the phase coherence did not show decremental patterns over higher % control conditions. The phase coherence was lowest in the 25% control condition, and highest in the 75% control condition (Fig 4).

In the gamma band, phase coherence changes were observed in the left frontal and right temporal areas (FP1-T6: *χ* ^2^ = 12.85 p = 0.012; Friedman test), the left frontal and right occipital areas (FP1-O2, *χ* ^2^ = 12.90p = 0.012; Friedman test), the left frontal and right temporal (F7-T4L: *χ* ^2^ = 9.65, p = 0.047), and the right frontal and mid central (F8-Cz: *χ* ^2^ = 9.65, p = 0.004; Friedman test) as % control conditions were changed. The phase coherence did not show gradual decline as higher % control conditions, but the weakest synchronization was in the 25% control condition (Fig 4C).

## Discussion

This study yields two major findings: (1) alpha frequency modulation was most clearly directly related to changes in SA; and (2) The anterior frontal lobe might be a hub for SA processing, because functional connection within the frontal lobes (FP2, F7, Fz) varied according to SA changes in alpha band. The frontal areas were also connected to the other areas (the middle central, parietal, temporal, and occipital lobes in the right hemisphere) during SA modulation in the other frequency bands (beta and gamma band).

Our study suggests that the alpha band may be the main oscillatory band relating to SA alterations. The degree of desynchronization was larger at higher levels of control (Fig 3B). A clear pattern of connectivity negatively linearly correlated with % control was only found in the alpha band (Fig 4A). The phase coherence became smaller as the virtual hand was more controlled. This inverse correlation seems to be consistent with a previous fMRI study in our lab. Brain hemodynamic response was lower in the 100% control condition compared to the other conditions [10]. These fMRI and EEG studies suggest that the system might be more active when there is a lack of agency or less agency. During normal circumstances, the self-agency system may not be vigorously activated and the brain just accepts that as being routine. However, when movement does not match what was willed, the agency system may be brought more into play. It is mismatch more than match that ordinarily becomes conscious. We think that the agency system is, of course, activated in the complete control condition, because the alpha band activity was different between 100% control and resting conditions. These findings seem to agree with the neural efficiency hypothesis that brain may tend to work efficiently with less activation in good performance [30, 31]. Previous EEG coherence studies also support this explanation [32, 33]. In a visuomotor tracking task, fronto-central coupling was increased with more tracking error [33]. They suggest that the alpha band contributes to effective functional integration of visuomotor areas. Cortico-cortical coupling was weaker in expert motor performance group than in novices [32].

The finding that there is a greater power decrease at the higher level of motor control may contradict a previous study. Larger desynchronization of the alpha band was observed with incongruent images of self-location than in congruent images [34]. Our findings of relative power changes might also contradict our phase coherence analyses, because the greater desynchronization in the better motor control suggests greater brain activation. As described before, the phase coherence findings suggest that there is less brain activation in better motor control. A possible explanation is that power changes and phase coherence may reflect different brain functions. Several studies show that local and global EEG activities of the alpha band can be separately manipulated with a motor task [35]. The synchronization between distant cortical areas in specific frequency bands may occur without corresponding changes in local oscillatory activity [21]. Local alpha oscillation modulates multiple sensory inputs and, with attention, selectively suppresses distracting information [36,37]. Alpha oscillations operate by suppressing irrelevant or distracting information that might interfere with performance [38–42]. Higher absolute power, that is, less desynchronization, might be associated with suppression of conflicting information. Another possible explanation is different study designs. The previous study used a simple, repetitive visuomotor task that employed a visuotactile conflict task with no movement [34].

At first glance, the beta band findings are similar to the results of the alpha band. The beta band desynchronization was greater with better motor control, and phase coherence was associated with SA changes. However, the desynchronization occurred in the left hemisphere (Fig 3C). The phase coherence did not show any parallel pattern over SA modulation. The changes of phase coherence were mainly in the fronto-occipital (or central-occipital) connections, suggesting that these connections might be related to visual information processing (Fig 4B).

Profound relative decreases of gamma band power were seen only in the 100% control condition. Unlike the alpha and beta band power, there was little or no relative power changes in the other conditions (Fig 3). Gamma band phase coherence was observed in each % control condition, but the connectivity did not correlate with SA (Fig 4C). Our results suggest that these beta and gamma frequency bands might not be directly related to SA, because of no correlation with the behavioral measurement (% control condition paradigm). These beta and gamma frequency bands might have a role in general information processing (i.e., non-specific to SA) of visuomotor control. Long-range cortical synchronization of these frequency bands is known to have an essential role in diverse cognitive processes (perception, attention, visuomotor integration, and visuomotor working memory) in multiple cortical regions [21,43–45]. A study also suggested that long-range beta band oscillations might be related to maintenance of the current situation while gamma band oscillations might be related to changes in the situation [46]. The other possible interpretation is that these frequency bands might be associated with unconscious components with SA. Several different levels of SA have been suggested [12,47]. These levels are postulated naturalistically: non-conceptual level (feeling of agency, implicit self-representation); conceptual level (judgment of agency, explicit self-representation); and meta-representation level (mental representation attributing to the self or to others) [47]. The non-conceptual level is thought to be associated with perception such as sensory feedback and the states of SA in the non-conceptual level might show non-analyzable processes. People might feel their SA only in the conceptual level [47].

Neural oscillations play an essential role in both local activity [48,49] and long-range communication [15,50]. There are many oscillatory bands in neuronal networks, ranging from 0.05 Hz to 500 Hz, existing simultaneously and temporally in the same or different brain structures, and communicating with each other [51]. Their physiological roles have been partially reported, but there are still many unknowns. In our study, each frequency band may have a different role [52], because the changes of the power spectrum and phase coherences over SA alteration looked dissimilar in all frequency bands, and the electrodes showing those changes may be different in each frequency band. Even in the same frequency band, the function of the local oscillatory activity might differ from the function of the long-range synchronization, because the cortical areas with the local oscillatory changes seemed to be different from the areas with the changes in long-range synchronization in some frequency bands such alpha band. As mentioned above, their different roles might be specific or non-specific to SA, and might be associated with different levels of SA [12,47], which remains to be elucidated in the future.

Areas showing significant changes of functional connectivity in response to the loss of SA seem to be consistent with earlier fMRI and PET studies [2,10]. The anatomical correlates of SA were previously reported on the basis of fMRI [2,5–10,53], but their function is not clearly understood [2,5,7–10,53,54]. The sensorimotor, parietal, and occipital areas are involved in processing sensory information related to movements and the frontal area is concerned with multimodal integration (input-output process) of the diverse sensory information [2,5,6,10,55–62]. The role of the temporal lobe may be similar to the role of the frontal area [63], and includes state assessment of self-movement during visually guided movement [10,59,63]. Our study shows that the fronto-central regions appear to be the main center to judge matching or mismatching of several types of input and output information [55,59]. Previous studies support this idea. A TMS study reported SA alteration after inhibition of the pre-supplementary motor area, but not after inhibition of the sensorimotor area [58]. This suggests that the pre-supplementary motor area has a role in SA. An fMRI experiment using a CyberGlove in our lab found two discrete sets of regions: earlier and later activated regions. The earlier activated regions seem to be related to mismatch identification, and the later activated regions including the bilateral frontal areas seem to be a receiver of this information and generator of SA [10].

Our study also shows that bilateral fronto-central regions seem to be functionally connected with the other cortical regions, supporting the idea that the fronto-central regions may be a central node receiving various types of information such as sensory information (somatic, visual, etc.) and state estimation between self-movements and perception, and adjusting motor output [55,59].

In summary, our data shows that alpha band is directly related to SA and the network communication of the alpha band in the anterior frontal area may be the main mechanism of SA, because there was a parallel relationship between the phase synchronization and SA modulation.

Our study has some limitations. Some recent studies suggest that personality differences might change the agency experience of volitional action [64–66]. Because we did not measure individual personality, we do not know whether personality differences might affect our results. However, we believe that this contribution might be very minimal in our study, because we carefully recruited healthy people after comprehensive interview, general medical screening, neurological examination, and brain MRI scan.

As we described earlier, we asked each participant to keep pace when performing the hand movements during the entire EEG recording, and tried to control the speed to be in the same range for all the participants, but we did not measure the speed to check. We assumed that the electrical activity at a given electrode reflects the cerebral activity just beneath it without taking volume conduction into account, and that each electrode is correctly located in each responsible anatomical region described in these results. Because we did not perform source analysis that would allow us to localize the source of the presented effects, these EEG results might not be sufficiently accurate to be easily compared to previous fMRI and PET studies. Spurious connectivity may be possible in some of our results due to volume conduction. Phase coherence could be explained by activity in a single cortical source being recorded at several electrodes. However, the reduction of volume conducted alpha activity with increased of SA found here, would lead to increased rather than decreased coherence within the frontal network.

## Supporting Information

S1 Table Electrodes showing significant spectral power changes. (DOC)

S2 Table Electrode pairs with significant phase coherence differences. (DOC)
