# Correction: Brain Networks Responsible for Sense of Agency: An EEG Study

**DOI:** 10.1371/journal.pone.0137769

**Published:** 2015-09-02

**Authors:** Suk Yun Kang, Chang-Hwan Im, Miseon Shim, Fatta B. Nahab, Jihye Park, Do-Won Kim, John Kakareka, Nathanial Miletta, Mark Hallett

In the Funding section, the grant number from the National Research Foundation of Korea is listed incorrectly. The correct grant number is NRF-2012R1A2A2A03045395.

The complete, correct Funding statement is: This work was supported by the intramural research program of the National Institute of Neurological Disorders and Stroke (NINDS) at the NIH and in part by the Public Welfare and Safety Research Program through the National Research Foundation of Korea (NRF) funded by the Ministry of Science, ICT & Future Planning (No. NRF-2012R1A2A2A03045395).
