# Supplementary material for: Brain Networks Responsible for Sense of Agency: An EEG Study
Source: PLoS One. 2015 Aug 13;10(8):e0135261. doi: 10.1371/journal.pone.0135261 (PMC4536203; doi:10.1371/journal.pone.0135261)
Supplement: S1 Table — (DOC) [file pone.0135261.s001.doc]

Table S1

**Absolute power spectrum**

| **Alpha** | | | | | | | | | | | | | | |
| --- | --- | --- | --- | --- | --- | --- | --- | --- | --- | --- | --- | --- | --- | --- |
| **0%** | | | **25%** | | | **50%** | | | **75%** | | | **100%** | | |
| **Channel** | **Power^*^** | **p-value** | **Channel** | **Power** | **p-value** | **Channel** | **Power** | **p-value** | **Channel** | **Power** | **p-value** | **Channel** | **Power** | **p-value** |
|  |  |  |  |  |  | C4 | 6.79 | 0.00023 | P3 | 4.83 | 0.00022 | P3 | 4.82 | 0.00014 |
|  |  |  |  |  |  | P3 | 4.99 | 0.00008 | P4 | 5.62 | 0.00012 | P4 | 5.37 | 0.00022 |
|  |  |  |  |  |  | P4 | 5.82 | 0.00011 | T6 | 6.91 | 0.00009 | T6 | 6.68 | 0.00010 |
|  |  |  |  |  |  | Pz | 4.03 | 0.00019 |  |  |  |  |  |  |
|  |  |  |  |  |  | T6 | 7.13 | 0.00011 |  |  |  |  |  |  |

* Power: mean value of absolute power spectrum

| **Alpha – absolute power in resting condition** | | | | | | | | | | | | | |
| --- | --- | --- | --- | --- | --- | --- | --- | --- | --- | --- | --- | --- | --- |
| **Channel** | C4 | P3 | P4 | Pz | T6 |  |  |  |  |  |  |  |  |
| **Power** | 3.70 | 2.09 | 2.58 | 1.25 | 3.91 |  |  |  |  |  |  |  |  |

| **Beta** | | | | | | | | | | | | | | |
| --- | --- | --- | --- | --- | --- | --- | --- | --- | --- | --- | --- | --- | --- | --- |
| **0%** | | | **25%** | | | **50%** | | | **75%** | | | **100%** | | |
| **Channel** | **Power** | **p-value** | **Channel** | **Power** | **p-value** | **Channel** | **Power** | **p-value** | **Channel** | **Power** | **p-value** | **Channel** | **Power** | **p-value** |
| C3 | 13.90 | 0.000004 | C3 | 13.81 | 0.000012 | C3 | 14.02 | 0.000001 | C3 | 13.96 | 0.000001 | C3 | 13.96 | 0.000001 |
| C4 | 14.88 | 0.000002 | C4 | 14.92 | 0.000007 | C4 | 15.05 | 0.000004 | C4 | 15.00 | 0.000000 | C4 | 15.00 | 0.000000 |
| Cz | 13.53 | 0.000018 | Cz | 13.46 | 0.000061 | Cz | 13.70 | 0.000007 | Cz | 13.61 | 0.000003 | Cz | 13.61 | 0.000003 |
| F3 | 12.90 | 0.000283 | Fz | 13.32 | 0.000110 | F3 | 13.04 | 0.000066 | F3 | 13.01 | 0.000088 | F3 | 13.01 | 0.000088 |
| Fz | 13.38 | 0.000017 | O1 | 13.33 | 0.000158 | F4 | 13.73 | 0.000038 | F4 | 13.60 | 0.000158 | F4 | 13.60 | 0.000158 |
| O1 | 13.40 | 0.000058 | P3 | 13.56 | 0.000002 | Fz | 13.57 | 0.000009 | Fz | 13.42 | 0.000007 | Fz | 13.42 | 0.000007 |
| P3 | 13.61 | 0.000001 | P4 | 14.52 | 0.000009 | O1 | 13.48 | 0.000003 | O1 | 13.49 | 0.000000 | O1 | 13.49 | 0.000000 |
| P4 | 14.51 | 0.000000 | Pz | 13.29 | 0.000055 | O2 | 14.46 | 0.000003 | O2 | 14.26 | 0.000002 | O2 | 14.26 | 0.000002 |
| Pz | 13.38 | 0.000002 | T5 | 13.56 | 0.000024 | P3 | 13.72 | 0.000000 | P3 | 13.71 | 0.000000 | P3 | 13.71 | 0.000000 |
| T6 | 15.22 | 0.000003 | T6 | 15.32 | 0.000004 | P4 | 14.70 | 0.000001 | P4 | 14.66 | 0.000000 | P4 | 14.66 | 0.000000 |
|  |  |  |  |  |  | Pz | 13.52 | 0.000001 | Pz | 13.43 | 0.000004 | Pz | 13.43 | 0.000004 |
|  |  |  |  |  |  | T5 | 13.62 | 0.000053 | T5 | 13.60 | 0.000039 | T3L | 13.60 | 0.000039 |
|  |  |  |  |  |  | T6 | 15.43 | 0.000001 | T6 | 15.41 | 0.000000 | T4L | 15.41 | 0.000000 |
|  |  |  |  |  |  |  |  |  |  |  |  | T5 | 13.96 | 0.000001 |
|  |  |  |  |  |  |  |  |  |  |  |  | T6 | 15.00 | 0.000000 |

| **Beta – absolute power in resting condition** | | | | | | | | | | | | | |
| --- | --- | --- | --- | --- | --- | --- | --- | --- | --- | --- | --- | --- | --- |
| **Channel** | C3 | C4 | Cz | F3 | F4 | Fz | O1 | O2 | P3 | P4 | Pz | T3L | T4L |
| **Power** | 12.39 | 13.39 | 12.37 | 12.16 | 12.69 | 12.42 | 12.67 | 13.36 | 12.43 | 13.23 | 12.27 | 13.15 | 15.80 |
| **Channel** | T5 | T6 |  |  |  |  |  |  |  |  |  |  |  |
| **Power** | 12.87 | 14.04 |  |  |  |  |  |  |  |  |  |  |  |

| **Gamma** | | | | | | | | | | | | | | |
| --- | --- | --- | --- | --- | --- | --- | --- | --- | --- | --- | --- | --- | --- | --- |
| **0%** | | | **25%** | | | **50%** | | | **75%** | | | **100%** | | |
| **Channel** | **Power** | **p-value** | **Channel** | **Power** | **p-value** | **Channel** | **Power** | **p-value** | **Channel** | **Power** | **p-value** | **Channel** | **Power** | **p-value** |
|  |  |  |  |  |  |  |  |  |  |  |  | Cz | 29.22 | 0.000045 |
|  |  |  |  |  |  |  |  |  |  |  |  | P3 | 29.53 | 0.000028 |
|  |  |  |  |  |  |  |  |  |  |  |  | Pz | 29.60 | 0.000029 |

| **Gamma – absolute power in resting condition** | | | | | | | | | | | | | |
| --- | --- | --- | --- | --- | --- | --- | --- | --- | --- | --- | --- | --- | --- |
| **Channel** | C3 | P3 | Pz |  |  |  |  |  |  |  |  |  |  |
| **Power** | 12.39 | 13.39 | 12.37 |  |  |  |  |  |  |  |  |  |  |
