# Supplementary material for: Brain Networks Responsible for Sense of Agency: An EEG Study
Source: PLoS One. 2015 Aug 13;10(8):e0135261. doi: 10.1371/journal.pone.0135261 (PMC4536203; doi:10.1371/journal.pone.0135261)
Supplement: S2 Table — (DOC) [file pone.0135261.s002.doc]

Table S2

**Phase Coherence**

| **Alpha** | | | | | | | | | | | | | | |
| --- | --- | --- | --- | --- | --- | --- | --- | --- | --- | --- | --- | --- | --- | --- |
| **0%** | | | **25%** | | | **50%** | | | **75%** | | | **100%** | | |
| **Channel** | **Coh**^**^ | **p-value** | **Channel** | **Coh** | **p-value** | **Channel** | **Coh** | **p-value** | **Channel** | **Coh** | **p-value** | **Channel** | **Coh** | **p-value** |
| C3_C4 | 0.64 | 0.000109 | FP1_C4 | 0.44 | 0.000082 | C3_C4 | 0.66 | 0.000050 | C3_C4 | 0.64 | 0.000213 | F7_F8 | 0.29 | 0.000092 |
| C3_FP2 | 0.51 | 0.000019 | FP1_P3 | 0.39 | 0.000291 | F3_C4 | 0.58 | 0.000193 | C3_FP2 | 0.50 | 0.000129 | F7_FP2 | 0.49 | 0.000035 |
| F3_C4 | 0.58 | 0.000022 | FP2_C4 | 0.50 | 0.000049 | FP1_P3 | 0.40 | 0.000230 | F3_C4 | 0.58 | 0.000155 | FP1_C3 | 0.59 | 0.000288 |
| F4_O1 | 0.27 | 0.000159 |  |  |  | FP2_P3 | 0.32 | 0.000202 | F7_FP2 | 0.50 | 0.000166 | FP1_P3 | 0.40 | 0.000074 |
| F8_P3 | 0.26 | 0.000161 |  |  |  | FP2_Pz | 0.34 | 0.000267 | FP1_C3 | 0.60 | 0.000014 | FP1_P4 | 0.29 | 0.000193 |
| F8_P4 | 0.30 | 0.000067 |  |  |  |  |  |  | FP1_C4 | 0.46 | 0.000022 | FP2_C4 | 0.51 | 0.000234 |
| F8_Pz | 0.30 | 0.000016 |  |  |  |  |  |  | FP1_P3 | 0.40 | 0.000050 | FP2_O1 | 0.22 | 0.000238 |
| FP1_C3 | 0.60 | 0.000042 |  |  |  |  |  |  | FP1_P4 | 0.30 | 0.000229 | FP2_P3 | 0.33 | 0.000002 |
| FP1_C4 | 0.45 | 0.000221 |  |  |  |  |  |  | FP2_C4 | 0.52 | 0.000007 | FP2_P4 | 0.31 | 0.000020 |
| FP1_O1 | 0.26 | 0.000024 |  |  |  |  |  |  | FP2_P3 | 0.32 | 0.000082 | FP2_Pz | 0.35 | 0.000032 |
| FP1_P3 | 0.40 | 0.000002 |  |  |  |  |  |  | FP2_P4 | 0.30 | 0.000048 | FZ_C4 | 0.68 | 0.000140 |
| FP2_C4 | 0.50 | 0.000282 |  |  |  |  |  |  | FP2_Pz | 0.34 | 0.000033 | FZ_F7 | 0.53 | 0.000107 |
| FP2_O1 | 0.22 | 0.000009 |  |  |  |  |  |  | Fz_C4 | 0.69 | 0.000157 |  |  |  |
| FP2_P3 | 0.34 | 0.000000 |  |  |  |  |  |  |  |  |  |  |  |  |
| FP2_Pz | 0.36 | 0.000001 |  |  |  |  |  |  |  |  |  |  |  |  |
| FP2_T3L | 0.28 | 0.000113 |  |  |  |  |  |  |  |  |  |  |  |  |
| FP2_T5 | 0.22 | 0.000047 |  |  |  |  |  |  |  |  |  |  |  |  |
| Fz_C4 | 0.68 | 0.000053 |  |  |  |  |  |  |  |  |  |  |  |  |
| Fz_O1 | 0.32 | 0.000046 |  |  |  |  |  |  |  |  |  |  |  |  |

**Coh: mean value of phase coherence

| **Alpha – phase coherence in resting condition** | | | | | | | | | | | | | |
| --- | --- | --- | --- | --- | --- | --- | --- | --- | --- | --- | --- | --- | --- |
| **Channel** | C3 _C4 | C3 _FP2 | F3 _C4 | F4 _O1 | F8 _P3 | F8 _P4 | F8 _PZ | FP1_C3 | FP1_C4 | FP1_O1 | FP1_P3 | FP2_C4 | FP2_O1 |
| **Coh** | 0.56 | 0.42 | 0.49 | 0.22 | 0.20 | 0.24 | 0.25 | 0.51 | 0.35 | 0.19 | 0.31 | 0.40 | 0.15 |
| **Channel** | FP2_P3 | FP2_PZ | FP2_T3L | FP2_T5 | FZ _C4 | FZ _O1 |  |  |  |  |  |  |  |
| **Coh** | 0.24 | 0.26 | 0.23 | 0.17 | 0.59 | 0.27 |  |  |  |  |  |  |  |

| **Beta** | | | | | | | | | | | | | | |
| --- | --- | --- | --- | --- | --- | --- | --- | --- | --- | --- | --- | --- | --- | --- |
| **0%** | | | **25%** | | | **50%** | | | **75%** | | | **100%** | | |
| **Channel** | **Coh** | **p-value** | **Channel** | **Coh** | **p-value** | **Channel** | **Coh** | **p-value** | **Channel** | **Coh** | **p-value** | **Channel** | **Coh** | **p-value** |
| C3_C4 | 0.56 | 0.000027 | C3_C4 | 0.57 | 0.000044 | C3_C4 | 0.57 | 0.000021 | C3_C4 | 0.57 | 0.000018 | C3_C4 | 0.57 | 0.000069 |
| C3_O1 | 0.55 | 0.000241 | C3_O2 | 0.43 | 0.000096 | C3_O1 | 0.55 | 0.000110 | C3_O1 | 0.55 | 0.000032 | C3_O1 | 0.54 | 0.000040 |
| C3_O2 | 0.44 | 0.000024 | C3_P4 | 0.51 | 0.000015 | C3_O2 | 0.44 | 0.000010 | C3_O2 | 0.45 | 0.000004 | C3_O2 | 0.43 | 0.000150 |
| C3_P4 | 0.52 | 0.000006 | C3_Pz | 0.67 | 0.000019 | C3_P3 | 0.77 | 0.000152 | C3_P4 | 0.52 | 0.000002 | C3_P4 | 0.51 | 0.000034 |
| C3_Pz | 0.67 | 0.000046 | C3_T6 | 0.31 | 0.000183 | C3_P4 | 0.52 | 0.000000 | C3_Pz | 0.67 | 0.000171 | C3_Pz | 0.68 | 0.000278 |
| C3_T4L | 0.30 | 0.000027 | C4_O1 | 0.41 | 0.000001 | C3_Pz | 0.68 | 0.000001 | C3_T4L | 0.31 | 0.000002 | C3_T4L | 0.30 | 0.000006 |
| C3T5 | 0.61 | 0.000137 | C4_O2 | 0.46 | 0.000002 | C3_T4L | 0.29 | 0.000280 | C3_T6 | 0.33 | 0.000004 | C3_T6 | 0.33 | 0.000002 |
| C3_T6 | 0.33 | 0.000001 | C4_P3 | 0.51 | 0.000044 | C3_T5 | 0.61 | 0.000187 | C4_O1 | 0.42 | 0.000003 | C4_O1 | 0.42 | 0.000045 |
| C4_O1 | 0.41 | 0.000253 | C4_Pz | 0.65 | 0.000114 | C3_T6 | 0.33 | 0.000001 | C4_O2 | 0.46 | 0.000000 | C4_O2 | 0.46 | 0.000018 |
| C4_O2 | 0.46 | 0.000279 | C4_T5 | 0.36 | 0.000027 | C4_O1 | 0.40 | 0.000004 | C4_P3 | 0.52 | 0.000019 | C4_P3 | 0.52 | 0.000068 |
| C4_P3 | 0.51 | 0.000099 | Cz_C4 | 0.76 | 0.000180 | C4_O2 | 0.46 | 0.000088 | C4_P4 | 0.71 | 0.000188 | C4_P4 | 0.71 | 0.000180 |
| C4_Pz | 0.64 | 0.000222 | Cz_O1 | 0.46 | 0.000149 | C4_P3 | 0.52 | 0.000005 | C4_Pz | 0.64 | 0.000227 | C4_Pz | 0.65 | 0.000044 |
| C4_T3L | 0.36 | 0.000250 | Cz_P4 | 0.62 | 0.000022 | C4_Pz | 0.65 | 0.000033 | C4_T5 | 0.36 | 0.000009 | C4_T5 | 0.36 | 0.000155 |
| C4_T5 | 0.36 | 0.000013 | Cz_Pz | 0.74 | 0.000248 | C4_T5 | 0.36 | 0.000000 | C4_T6 | 0.48 | 0.000253 | Cz_O1 | 0.47 | 0.000130 |
| Cz_C4 | 0.76 | 0.000066 | Cz_T5 | 0.44 | 0.000037 | Cz_C3 | 0.77 | 0.000117 | Cz_C3 | 0.77 | 0.000210 | Cz_O2 | 0.44 | 0.000093 |
| Cz_O1 | 0.48 | 0.000072 | Cz_T6 | 0.36 | 0.000108 | Cz_C4 | 0.77 | 0.000090 | Cz_C4 | 0.76 | 0.000061 | Cz_P4 | 0.63 | 0.000002 |
| Cz_O2 | 0.45 | 0.000011 | F3_F7 | 0.57 | 0.000076 | Cz_O1 | 0.47 | 0.000041 | Cz_O1 | 0.48 | 0.000003 | Cz_Pz | 0.75 | 0.000015 |
| Cz_P3 | 0.65 | 0.000076 | F3_O1 | 0.33 | 0.000149 | Cz_O2 | 0.45 | 0.000061 | Cz_O2 | 0.46 | 0.000003 | Cz_T6 | 0.38 | 0.000001 |
| Cz_P4 | 0.63 | 0.000002 | F3_O2 | 0.28 | 0.000049 | Cz_P3 | 0.66 | 0.000010 | Cz_P3 | 0.66 | 0.000021 | F3_O1 | 0.34 | 0.000275 |
| Cz_Pz | 0.74 | 0.000032 | F3_P3 | 0.48 | 0.000059 | Cz_P4 | 0.63 | 0.000002 | Cz_P4 | 0.64 | 0.000000 | F3_O2 | 0.28 | 0.000028 |
| Cz_T5 | 0.45 | 0.000064 | F3_P4 | 0.36 | 0.000110 | Cz_Pz | 0.74 | 0.000036 | Cz_Pz | 0.74 | 0.000210 | F3_P4 | 0.37 | 0.000132 |
| Cz_T6 | 0.38 | 0.000010 | F3_Pz | 0.45 | 0.000050 | Cz_T5 | 0.45 | 0.000005 | Cz_T4L | 0.38 | 0.000001 | F3_T4L | 0.24 | 0.000227 |
| F3_O1 | 0.35 | 0.000002 | F3_T5 | 0.38 | 0.000013 | Cz_T6 | 0.38 | 0.000001 | Cz_T5 | 0.46 | 0.000011 | F3_T6 | 0.24 | 0.000045 |
| F3_O2 | 0.29 | 0.000000 | F4_O1 | 0.25 | 0.000163 | F3_O1 | 0.34 | 0.000004 | Cz_T6 | 0.39 | 0.000001 | F4_O1 | 0.26 | 0.000286 |
| F3_P3 | 0.49 | 0.000013 | F4_O2 | 0.24 | 0.000106 | F3_O2 | 0.29 | 0.000003 | F3_O1 | 0.34 | 0.000014 | F4_O2 | 0.25 | 0.000059 |
| F3_P4 | 0.37 | 0.000005 | F8_T5 | 0.18 | 0.000151 | F3_P3 | 0.49 | 0.000001 | F3_O2 | 0.29 | 0.000007 | F8_O1 | 0.18 | 0.000251 |
| F3_Pz | 0.46 | 0.000094 | FP1_F3 | 0.57 | 0.000188 | F3_P4 | 0.37 | 0.000005 | F3_P3 | 0.48 | 0.000197 | FP2_O1 | 0.20 | 0.000228 |
| F3_T3L | 0.45 | 0.000008 | FP1_F7 | 0.59 | 0.000234 | F3_Pz | 0.46 | 0.000007 | F3_P4 | 0.37 | 0.000005 | FP2_O2 | 0.19 | 0.000159 |
| F3_T4L | 0.24 | 0.000059 | FP1_T4L | 0.19 | 0.000285 | F3_T5 | 0.40 | 0.000001 | F3_T4L | 0.25 | 0.000019 | Fz_O1 | 0.33 | 0.000107 |
| F3_T5 | 0.40 | 0.000000 | FP1_T5 | 0.29 | 0.000215 | F3_T6 | 0.24 | 0.000005 | F3_T5 | 0.39 | 0.000028 | Fz_O2 | 0.29 | 0.000065 |
| F3_T6 | 0.24 | 0.000007 | FP2_O1 | 0.21 | 0.000112 | F4_O1 | 0.26 | 0.000029 | F3_T6 | 0.24 | 0.000015 | Fz_P4 | 0.43 | 0.000103 |
| F4_O1 | 0.26 | 0.000236 | FP2_T4L | 0.20 | 0.000177 | F4_O2 | 0.26 | 0.000023 | F4_O1 | 0.27 | 0.000006 | Fz_Pz | 0.50 | 0.000288 |
| F4_O2 | 0.25 | 0.000139 | FP2_T5 | 0.24 | 0.000036 | F4_P3 | 0.37 | 0.000217 | F4_O2 | 0.26 | 0.000003 | Fz_T4L | 0.29 | 0.000265 |
| F4_P4 | 0.39 | 0.000077 | Fz_O1 | 0.31 | 0.000181 | F4_P4 | 0.39 | 0.000009 | F4_P4 | 0.39 | 0.000031 | Fz_T6 | 0.26 | 0.000094 |
| F7_O2 | 0.25 | 0.000017 | Fz_O2 | 0.28 | 0.000047 | F4_Pz | 0.41 | 0.000074 | F4_Pz | 0.41 | 0.000233 |  |  |  |
| F7_P4 | 0.27 | 0.000242 | Fz_P3 | 0.46 | 0.000067 | F4_T5 | 0.27 | 0.000020 | F4_T4L | 0.30 | 0.000136 |  |  |  |
| F7_T5 | 0.40 | 0.000255 | Fz_P4 | 0.42 | 0.000011 | F4_T6 | 0.25 | 0.000210 | F4_T5 | 0.27 | 0.000041 |  |  |  |
| F8_O1 | 0.18 | 0.000098 | Fz_Pz | 0.49 | 0.000023 | F7_C4 | 0.34 | 0.000257 | F4_T6 | 0.26 | 0.000142 |  |  |  |
| F8_T5 | 0.18 | 0.000264 | Fz_T4L | 0.28 | 0.000155 | F7_O1 | 0.33 | 0.000002 | F7_O1 | 0.32 | 0.000024 |  |  |  |
| FP1_O1 | 0.25 | 0.000027 | Fz_T5 | 0.34 | 0.000066 | F7_O2 | 0.27 | 0.000000 | F7_O2 | 0.26 | 0.000001 |  |  |  |
| FP1_O2 | 0.21 | 0.000008 | Fz_T6 | 0.25 | 0.000088 | F7_P3 | 0.42 | 0.000057 | F7_P4 | 0.27 | 0.000011 |  |  |  |
| FP1_P4 | 0.26 | 0.000041 |  |  |  | F7_P4 | 0.28 | 0.000002 | F7_T5 | 0.41 | 0.000044 |  |  |  |
| FP1_T4L | 0.19 | 0.000002 |  |  |  | F7_Pz | 0.35 | 0.000157 | F8_O1 | 0.18 | 0.000059 |  |  |  |
| FP1_T5 | 0.31 | 0.000045 |  |  |  | F7_T4L | 0.20 | 0.000083 | F8_P4 | 0.24 | 0.000273 |  |  |  |
| FP2_F8 | 0.51 | 0.000082 |  |  |  | F7_T5 | 0.41 | 0.000007 | F8_T3L | 0.20 | 0.000049 |  |  |  |
| FP2_O1 | 0.21 | 0.000052 |  |  |  | F7_T6 | 0.22 | 0.000066 | F8_T4L | 0.25 | 0.000113 |  |  |  |
| FP2_P4 | 0.25 | 0.000027 |  |  |  | FP1_C4 | 0.37 | 0.000055 | F8_T5 | 0.18 | 0.000016 |  |  |  |
| FP2_T4L | 0.21 | 0.000002 |  |  |  | FP1_O1 | 0.26 | 0.000000 | FP1_O1 | 0.25 | 0.000018 |  |  |  |
| FP2_T5 | 0.23 | 0.000112 |  |  |  | FP1_O2 | 0.22 | 0.000015 | FP1_O2 | 0.21 | 0.000068 |  |  |  |
| Fz_O1 | 0.33 | 0.000006 |  |  |  | FP1_P3 | 0.35 | 0.000013 | FP1_P4 | 0.25 | 0.000009 |  |  |  |
| Fz_O2 | 0.30 | 0.000002 |  |  |  | FP1_P4 | 0.27 | 0.000001 | FP1_T4L | 0.20 | 0.000001 |  |  |  |
| Fz_P3 | 0.47 | 0.000028 |  |  |  | FP1_Pz | 0.32 | 0.000017 | FP1_T5 | 0.30 | 0.000026 |  |  |  |
| Fz_P4 | 0.43 | 0.000007 |  |  |  | FP1_T4L | 0.20 | 0.000001 | FP2_O1 | 0.21 | 0.000006 |  |  |  |
| Fz_P | 0.50 | 0.000089 |  |  |  | FP1_T5 | 0.31 | 0.000000 | FP2_O2 | 0.19 | 0.000053 |  |  |  |
| Fz_T3L | 0.40 | 0.000003 |  |  |  | FP2_O1 | 0.21 | 0.000017 | FP2_P3 | 0.27 | 0.000178 |  |  |  |
| Fz_T4L | 0.28 | 0.000179 |  |  |  | FP2_P4 | 0.25 | 0.000021 | FP2_P4 | 0.24 | 0.000029 |  |  |  |
| Fz_T5 | 0.35 | 0.000001 |  |  |  | FP2_T4L | 0.21 | 0.000004 | FP2_T4L | 0.21 | 0.000006 |  |  |  |
| Fz_T6 | 0.27 | 0.000039 |  |  |  | FP2_T5 | 0.23 | 0.000004 | FP2_T5 | 0.23 | 0.000013 |  |  |  |
| P3_T6 | 0.42 | 0.000103 |  |  |  | Fz_C4 | 0.65 | 0.000171 | Fz_O1 | 0.33 | 0.000002 |  |  |  |
| T3L_T4L | 0.27 | 0.000268 |  |  |  | Fz_O1 | 0.32 | 0.000016 | Fz_O2 | 0.30 | 0.000004 |  |  |  |
| T3L_T6 | 0.31 | 0.000054 |  |  |  | Fz_O2 | 0.30 | 0.000022 | Fz_P3 | 0.48 | 0.000082 |  |  |  |
| T5_T4L | 0.29 | 0.000097 |  |  |  | Fz_P3 | 0.47 | 0.000005 | Fz_P4 | 0.43 | 0.000002 |  |  |  |
|  |  |  |  |  |  | Fz_P4 | 0.43 | 0.000004 | Fz_Pz | 0.50 | 0.000093 |  |  |  |
|  |  |  |  |  |  | Fz_Pz | 0.50 | 0.000003 | Fz_T4L | 0.29 | 0.000005 |  |  |  |
|  |  |  |  |  |  | Fz_T5 | 0.35 | 0.000002 | Fz_T5 | 0.35 | 0.000010 |  |  |  |
|  |  |  |  |  |  | Fz_T6 | 0.26 | 0.000005 | Fz_T6 | 0.27 | 0.000020 |  |  |  |
|  |  |  |  |  |  | T5_P4 | 0.44 | 0.000152 | P3_O2 | 0.61 | 0.000237 |  |  |  |
|  |  |  |  |  |  |  |  |  | P3_P4 | 0.61 | 0.000103 |  |  |  |
|  |  |  |  |  |  |  |  |  | P3_T4L | 0.35 | 0.000045 |  |  |  |
|  |  |  |  |  |  |  |  |  | P3_T6 | 0.41 | 0.000105 |  |  |  |
|  |  |  |  |  |  |  |  |  | T3L_T4L | 0.27 | 0.000009 |  |  |  |
|  |  |  |  |  |  |  |  |  | T3L_T6 | 0.31 | 0.000057 |  |  |  |
|  |  |  |  |  |  |  |  |  | T5_P4 | 0.45 | 0.000245 |  |  |  |
|  |  |  |  |  |  |  |  |  | T5_T4L | 0.29 | 0.000039 |  |  |  |

| **Beta – phase coherence in resting condition** | | | | | | | | | | | | | |
| --- | --- | --- | --- | --- | --- | --- | --- | --- | --- | --- | --- | --- | --- |
| **Channel** | C3_C4 | C3_O1 | C3_O2 | C3_P4 | C3_Pz | C3_T4L | C3_T6 | C4_O1 | C4_O2 | C4_P3 | C4_P4 | C4_Pz | C4_T5 |
| **Coh** | 0.49 | 0.50 | 0.38 | 0.45 | 0.63 | 0.25 | 0.26 | 0.36 | 0.41 | 0.45 | 0.68 | 0.60 | 0.30 |
| **Channel** | C4_T6 | Cz_C3 | Cz_C4 | Cz_O1 | Cz_O2 | Cz_P3 | Cz_P4 | Cz_Pz | Cz_T4L | Cz_T5 | Cz_T6 | F3_O1 | F3_O2 |
| **Coh** | 0.44 | 0.72 | 0.72 | 0.42 | 0.39 | 0.61 | 0.58 | 0.71 | 0.34 | 0.41 | 0.32 | 0.28 | 0.22 |
| **Channel** | F3_P3 | F3_P4 | F3_T4L | F3_T5 | F3_T6 | F4_O1 | F4_O2 | F4_P4 | F4_Pz | F4_T4L | F4_T5 | F4_T6 | F7_O1 |
| **Coh** | 0.43 | 0.29 | 0.19 | 0.34 | 0.18 | 0.20 | 0.19 | 0.33 | 0.36 | 0.25 | 0.22 | 0.20 | 0.27 |
| **Channel** | F7_O2 | F7_P4 | F7_T5 | F8_O1 | F8_P4 | F8_T3L | F8_T4L | F8_T5 | FP1_O1 | FP1_O2 | FP1_P4 | FP1_T4L | FP1_T5 |
| **Coh** | 0.20 | 0.21 | 0.36 | 0.13 | 0.19 | 0.16 | 0.20 | 0.14 | 0.19 | 0.15 | 0.19 | 0.15 | 0.25 |
| **Channel** | FP2_O1 | FP2_O2 | FP2_P3 | FP2_P4 | FP2_T4L | FP2_T5 | Fz_O1 | Fz_O2 | Fz_P3 | Fz_P4 | Fz_Pz | Fz_T4L | Fz_T5 |
| **Coh** | 0.15 | 0.14 | 0.22 | 0.18 | 0.15 | 0.18 | 0.26 | 0.22 | 0.41 | 0.35 | 0.44 | 0.23 | 0.29 |
| **Channel** | Fz_T6 | P3_O2 | P3_P4 | P3_T4L | P3_T6 | T3L_T4L | T3L_T6 | T5_P4 | T5_T4L | Fz_T6 | P3_O2 | P3_P4 | P3_T4L |
| **Coh** | 0.20 | 0.57 | 0.57 | 0.31 | 0.37 | 0.23 | 0.27 | 0.41 | 0.26 | 0.20 | 0.57 | 0.57 | 0.31 |

| **Gamma** | | | | | | | | | | | | | | |
| --- | --- | --- | --- | --- | --- | --- | --- | --- | --- | --- | --- | --- | --- | --- |
| **0%** | | | **25%** | | | **50%** | | | **75%** | | | **100%** | | |
| **Channel** | **Coh** | **p-value** | **Channel** | **Coh** | **p-value** | **Channel** | **Coh** | **p-value** | **Channel** | **Coh** | **p-value** | **Channel** | **Coh** | **p-value** |
| Fz_Pz | 0.58 | 0.000179 |  |  |  | F7_O2 | 0.3 | 0.000177 | Cz_F8 | 0.29 | 0.000292 | F8_P3 | 0.23 | 0.000078 |
|  |  |  |  |  |  | F8_O2 | 0.22 | 0.000285 | F3_Pz | 0.5 | 0.000047 | F8_T3L | 0.21 | 0.000265 |
|  |  |  |  |  |  | FP1_O2 | 0.27 | 0.000151 | F7_C4 | 0.32 | 0.00024 | F8_T5 | 0.21 | 0.000059 |
|  |  |  |  |  |  | FP1_P4 | 0.29 | 0.000031 | F7_Cz | 0.37 | 0.000189 |  |  |  |
|  |  |  |  |  |  | FP1_Pz | 0.32 | 0.00012 | F7_P3 | 0.38 | 0.0002 |  |  |  |
|  |  |  |  |  |  | FP1_T4L | 0.24 | 0.000045 | F7_P4 | 0.3 | 0.000021 |  |  |  |
|  |  |  |  |  |  | FP1_T6 | 0.25 | 0.000024 | F7_Pz | 0.34 | 0.000069 |  |  |  |
|  |  |  |  |  |  | FP2_O2 | 0.25 | 0.000168 | F7_T4L | 0.24 | 0.000128 |  |  |  |
|  |  |  |  |  |  | FP2_T6 | 0.23 | 0.000125 | F8_O1 | 0.2 | 0.000079 |  |  |  |
|  |  |  |  |  |  |  |  |  | F8_P3 | 0.24 | 0.000053 |  |  |  |
|  |  |  |  |  |  |  |  |  | F8_Pz | 0.25 | 0.000122 |  |  |  |
|  |  |  |  |  |  |  |  |  | F8_T5 | 0.21 | 0.000226 |  |  |  |
|  |  |  |  |  |  |  |  |  | FP1_O1 | 0.27 | 0.00016 |  |  |  |
|  |  |  |  |  |  |  |  |  | FP1_P3 | 0.33 | 0.000264 |  |  |  |
|  |  |  |  |  |  |  |  |  | FP1_Pz | 0.31 | 0.000164 |  |  |  |
|  |  |  |  |  |  |  |  |  | FP2_O1 | 0.23 | 0.000143 |  |  |  |

| **Gamma – phase coherence in resting condition** | | | | | | | | | | | | | |
| --- | --- | --- | --- | --- | --- | --- | --- | --- | --- | --- | --- | --- | --- |
| **Channel** | Cz_F8 | F3_Pz | F7_C4 | F7_Cz | F7_P3 | F7_P4 | F7_Pz | F7_T4L | F7_O2 | F8_O1 | F8_O2 | F8_P3 | F8_Pz |
| **Coh** | 0.24 | 0.46 | 0.28 | 0.33 | 0.35 | 0.26 | 0.30 | 0.20 | 0.20 | 0.16 | 0.17 | 0.19 | 0.20 |
| **Channel** | F8_T5 | F8_T5 | FP1_O1 | FP1_O2 | FP1_P3 | FP1_P4 | FP1_Pz | FP2_O1 | FP2_T6 |  |  |  |  |
| **Coh** | 0.17 | 0.17 | 0.23 | 0.20 | 0.29 | 0.23 | 0.26 | 0.19 | 0.19 |  |  |  |  |
